# Remodeling the tumor microenvironment by oncolytic viruses: beyond oncolysis of tumor cells for cancer treatment

**DOI:** 10.1136/jitc-2021-004167

**Published:** 2022-05-31

**Authors:** Lihong Wang, Louisa S Chard Dunmall, Zhenguo Cheng, Yaohe Wang

**Affiliations:** 1 National Centre for International Research in Cell and Gene Therapy, Sino-British Research Centre for Molecular Oncology, State Key Laboratory of Esophageal Cancer Prevention and Treatment, School of Basic Medical Sciences, Academy of Medical Sciences, Zhengzhou University, Zhengzhou, China; 2 Centre for Cancer Biomarkers and Biotherapeutics, Barts Cancer Institute, Queen Mary University of London, London, UK

**Keywords:** oncolytic viruses, immunotherapy, tumor microenvironment, oncolytic virotherapy, translational medical research

## Abstract

Tumor cells manipulate the local environment in which they grow, creating a tumor microenvironment (TME) that promotes tumor survival and metastasis. The TME is an extremely complex environment rich in immunosuppressive cells and cytokines. Various methods to therapeutically target the complicated TME are emerging as a potential approach for cancer treatment. Oncolytic viruses (OVs) are one of the most promising methods for remodeling the TME into an antitumor environment and can be used alone or in combination with other immunotherapy options. OVs replicate specifically in tumor cells and can be genetically engineered to target multiple elements of the TME simultaneously, thus representing a therapeutic with the potential to modify the TME to promote activation of antitumor immune cells and overcome tumor therapeutic resistance and recurrence. In this review, we analyze the tropism of OVs towards tumor cells and explore the interaction between OVs and immune cells, tumor stroma, vasculature and the metabolic environment in detail to help understand how OVs may be one of our most promising prospects for long-term curative therapies. We also discuss some of the challenges associated with TME therapies, and future perspectives in this evolving field.

## Introduction

The health and economic burdens imposed by cancer are increasing significantly year on year and while traditional therapeutic approaches may offer successful curative options, urgent improvements in therapeutic options are required to address the increasing burden. Our growing knowledge regarding the interactions between malignant cells and the body is opening up promising avenues for the development of novel, multimodal approaches that target this dynamic. One of the most significant advances stems from our understanding that cancer is not comprised solely of malignant cells, rather cancers represent complex networks of transformed cells, non-transformed cells and soluble mediators that together make up the tumor microenvironment (TME).^1^ The TME provides the perfect niche for the developing tumor, providing a structural scaffold for malignant and metastatic cells via the proteinaceous extracellular matrix (ECM), creating a barrier that prevents entry of endogenous or exogenous antitumor therapeutics, actively supporting tumor growth through systems of signal mediators and supporting generation and maintenance of cancer stem cells, which lie at the root of cancer growth.^2^ Increasing understanding of this niche has revealed that therapeutic interventions must not rely on sole targeting of malignant cells, but should also actively target these supportive complexes.^3^

Oncolytic viruses (OVs) are immunotherapeutic agents with multiple mechanisms of action. They not only selectively kill cancer cells through direct oncolysis, but also target multiple elements of the TME simultaneously to induce tumor cell death and long-term immune activation. They are showing strong potential in pre-clinical and clinical settings as potent immune stimulants and can synergize effectively with other therapies to improve clinical outcomes. This review provides a comprehensive analysis of the changes within the TME induced by OV activity and highlights the challenges that must be addressed in order to realize their full clinical potential.

## Tumor-selective OV therapy

The link between cancer regression and viral infection has been noted for over a century. In 1904, Dock reported in the spontaneous regression of leukemia in a patient with co-incidental infection with influenza.^4^ The earliest trials involving use of viruses to eliminate tumor cells demonstrated mixed results and significant side effects due to the use of live, wild-type virus strains.^5^ The emergence of gene editing techniques and improved understanding of the nature of the TME, has led to a resurgence of interest in OV. To date, four OVs have been approved for treatment of cancer and many more are being investigated through late stage clinical trials in a variety of tumor types (table 1).

**Table 1:** OVs and their effect on the solid tumor microenvironment

Therapy	Virus type	Deletion/payloads	Effect on TME	Cancer models	Reference
VACV ΔF4L∆J2R	Vaccinia virus (double stranded DNA genome)	*F4L* and *J2R* deletions.	Tumor‐specific CD8^+^ T cells upregulation.	Bladder cancer	Potts *et al* 20
VVΔTKΔN1L		*TK* and *N1L deletion.*	Increased intratumoral CD4^+^ and CD8^+^T cells; Increased systemic NK cells; Neutrophil accumulation; increased IL-α, IL1-β and GCSF.	Lung cancer; Pancreatic cancer	Ahmed *et al* 23
VVΔTKΔN1L-IL21		*TK* and *N1L* deletion; *IL-21* payload.	Increased I.T CD8^+^T cell and CD8^+^ TCM; Enhanced circulating NK cells; Macrophage polarization to M1.	Pancreatic cancer;	Marelli *et al* 34
vvDD-IL-2-FPTM/FG/RG		*TK* and *VGF* deletion; membrane-bound *IL-2* payload.	TNF-α; increased CD4^+^Foxp3^–^, CD8^+^IFN-γ^+^ T cells, decreased PD-1^+^CTLA-4^+^CD8^+^, PD-1^+^Tim-3^+^CD8^+^, PD-1^+^TIGIT^+^CD8^+^, and PD-1^+^LAG-3^+^CD8^+^ T cells.	Ovarian cancer; Lung cancer; Colon cancer	Liu *et al* 29
vvCCL5		Western reserve strain, *TK* and *VGF* gene deletions, *CCL5* expression in TK under p7.5 promoter.	Increased levels of CD4^+^ T cells, DC (CD11c^+^) and NK cells; Increased IL-4 and IL-5 expression.	Colon cancer	Li *et al* 38
vvTK-IL-36*γ*, vvDD-IL-36*γ*, vvTD-IL-36*γ*		Insertion of an active form of *IL-36*γ into three VV backbones with different tumor selectivity and oncolytic activities.	Increased levels of tumor antigen-specific CD4^+^ and CD8^+^ T cells.	Colon cancer	Yang *et al* 35
GLV-1h68		Renilla luciferase Aequoria-GFP fusion protein, β-galactosidase and β-glucoronidase inserted into F14.5 L, J2R (TK) and A56R loci of Lister strain virus.	Increased IFN-γ, IP-10, MCP-1, MCP-3, MCP-5, RANTES and TNF-γ; A greater infiltration of macrophages and NK cells.	Colorectal cancer	Ehrig *et al* 41
GLV-1h107, GLV-1h108, GLV-1h109		*scAb GLAF-1* inserted into the J2R (TK) locus under SE, SEL, and SL promoters (respectively) in a Lister strain virus.	Decreased microvessel density.	Lung cancer	Frentzen *et al* 95
LIVP1.1.1		A less virulent wild-type isolate of a strongly replicating LIVP (Lister) strain.	CD11b^+^ ly6G^+^ MDSCs recruitment, enhanced iNOS expression; higher production of NO.	Colon cancer	Kilinc *et al* 61
VV.HPGD		Expression of *HPGD* from *TK* deleted Western Reserve strain.	Induction of a more rapid and robust reduction in MDSC and TReg.	Renca tumor; Colorectal cancer	Hou *et al* 64
OVV-CXCR4-A-mFc		Expression of a CXCR4 antagonist from a *TK-VGF-*deleted Western reserve strain virus.	Destruction of I.T microvessels, lower accumulation of circulating endothelial progenitor cells and neutrophils/granulocytic-MDSCs, enhanced vaccinia-mediated activation of antitumor antibody responses.	Breast cancer	Gil *et al* 65
EphA2-TEA-VV		vvDD armed with bispecific T-cell engager that bind both to human CD3 and tumor cell surface antigen EphA2.	Activation of T cells; Increased IFN-γ and IL-2 secretion.	Lung cancer	Yu *et al* 81
JX-594		Disruption of the viral *TK* gene and expression of the *hGM-CSF and β-gal* transgenes under control of the synthetic early-late and p7.5 promoters in the Wyeth strain virus.	Infection and transgene expression in tumor cells and VEGF- and FGF-2–activated human endothelial cells. Vascular collapse.	Breast cancer	Breitbach *et al*; Breitbach *et al* 88 89
mpJX-594		Mouse-prototype JX-594.	Vascular pruning and prolong leakage in tumors; Widespread CD8^+^T cell dependent tumor cell killing.	Glioma	Kim *et al* 90
GLV-1h164		Single-chain antibody sequence for GLAF-2, an antibody directed against VEGF, inserted into Lister strain, *F14.5L/J2R/A56R*-deleted virus.	Inhibition of vascularization.	Breast cancer	Gholami *et al* 96
VV^leptin^		*Leptin*-armed VV WR strain with *TK* deletion.	Leptin-induced metabolic support allows immune cells to be polyfunctional, proliferative, and mediate tumor control; decreased TRegs; increased KLRG1^hi^CD127^+^ memory precursors.	Melanoma	Rivadeneira *et al* 106
VVhEA		Lister strain, *TK* deletion. Armed with *endostatin-angiostatin* fusion gene.	Inhibition of angiogenesis; Reversal of VEGF-induced cancer-associated systemic syndrome.	Pancreatic cancer	Tysome *et al* 91
HSV1716	Herpes simplex virus (double stranded DNA genome)	Deletion in the *RL1* genes encoding *ICP34.5.*	Increased CD11b^+^ Ly6C^+^ monocytes, CD11b^+^, LY6G^+^ neutrophils, CD3^+^ T cells, and CD8^+^ cytotoxic T cells; reduced TRegs; TAMs reprogramming.	Breast cancer	Kwan *et al* 47
		Deletion or mutation of the *RL1* gene.	An early influx of immune cells, mainly of CD4^+^ T cells, NK cells, and macrophages.	Melanoma	Miller and Fraser^51^
G207		Deletions of both *γ34.5 loci* and a *LacZ* insertion disabling the *UL39* gene.	Increase GADD34 expression in hypoxic cells.	Glioblastoma	Aghi *et al* 98
TVEC (HSV-GM-CSF)		Deletion of the *ICP34.5 and ICP47* genes. Insertion of *GM-CSF.*	Control of M1/M2 ratios MART-1 specific CD8^+^ T cell accumulation, TReg and MDSC decreased.	pancreatic cancer Melanoma	Liu *et al* Kohlhapp and Kaufman^15 44^
JS1/34.5−/47−/GM-CSF		*ICP34.5 and ICP47* gene deletion; insertion of human or mouse *GM-CSF.*	Increased MHC I expression.	Breast cancer	Liu *et al* 29
G47Δ-mIL12		Deletion of *α47 and γ34.5* genes and insertion of *LacZ into ICP6*; Insertion of mouse *IL-12* into *ICP6* gene.	Increase CD45^+^ immune cells and CD8^+^ T cells; macrophages infiltration, granulocytic and monocytic MDSC reduction, TReg reduction, DC trafficking to spleen.	Triple-negative breast cancer	Ghouse *et al* 32
G47Δ-mIL12		Deletion of *α47 and γ34.5* genes and insertion of *LacZ* into *ICP6*; Insertion of mouse *IL-12* into *ICP6* gene.	Reduction of TRegs, stimulation of Th1-type immunity, T cell mediated survival advantage; inhibition of tumor angiogenesis.	Glioblastoma	Cheema *et al* 69
VAE		*γ34.5 and ICP6* deleted HSV-1 carrying the *endostatin–angiostatin fusion* gene.	Reduced microvessel density.	Glioblastoma	Zhang *et al* 92
VSV	Vesicular stomatitis virus (negative-strand RNA genome)	-	Production of IL-28; Recruitment of CD11b^+^ GR1^+^ cells and plasmacytoid DC; Sensitized tumors to NK cell recognition and killing.	Melanoma	Wongthida *et al* 54
			Reduces CD31 expression; destruction of tumor vasculature.	Colon cancer	Breitbach *et al* 89
			Infection and killing of hypoxic cancer cells.	Glioblastoma	Connor *et al* 99
			CXCL1 and CXCL5 activation; Loss of blood flow to the interior of the tumor induced by neutrophil accumulation.	Colon cancer	Breitbach *et al* 57
			Viral transduced MDSCs can switch from the M2 phenotype to M1.	Colon cancer	Eisenstein *et al* 63
			Generalized T-cell activation; Increased IFN-γ.	Mesothelioma	Willmon *et al* 60
rVSV-UL141		VSV expressing cytomegalovirus *UL141* to downregulate *CD155*	Inhibition of NK recruitment; Decreased I.T accumulations of NK and NKT.	Hepatocellular carcinoma	Altomonte *et al* 56
VSV-IFNβ		VSV expressing the *IFN-β* gene	Stimulation of NK, cytotoxic T cells and DC activity.	Cervical cancer; Mammary adenocarcinoma; Prostatic cancer	Obuchi *et al* 36
Reovirus	Reovirus (double-stranded RNA genome)	Combination with surgery	Increased CD69a and tetherin expression and *IFIT1* mRNA expression in NK cells; Activation of NK cells.	Colorectal cancer	El-Sherbiny *et al* 52
			Reduced SLC2A1, ABCB1, MMP2, twist family bHLH transcription factor 1, and VEGFA expression in tumor cells; downregulation of HIF-1α.	Lung cancer	Hotani *et al* 100
			Inhibition of suppressive activity of MDSCs; increased IFN-α, IFN-β, IL-6, and IL-12β expression.	Melanoma	Katayama *et al* 62
MeV and MuV	Paramyxo viruses (negative-strand RNA genomes)		Induction of macrophage to an anti-tumor M1 phenotype.	Breast cancer	Tan *et al* 46
MV-aCTLA-4; MV-aPD-L1	Measles virus (negative-strand RNA genomes)	MV Edmonston strain encoding antibodies against CTLA-4 or PD-L1.	Increased in CD3^+^ T cells; decreased in TRegs; Increased CD8^+^/TReg ratios and IFN-γ expression.	Melanoma	Engeland *et al* 72
MV-hE:A and MV-mE:A		MV Edmonston strain encoding endostatin and angiostatin	Inhibition of angiogenic factors and blood vessel formation	Medulloblastoma	Hutzen *et al* 93
MV-m-uPA MV-h-uPA		MV Edmonston strain expressing murine or human *uPAR.*	Simultaneous infection of stromal and tumor cells; Upregulation of Fez1 and Pycard; Downregulation of DLL-4, Angiopoietin 2, PECAM, Tie-1, and FOS EGR2, CREB-5, IL-6, Map2K1, CCL22, TIMD4 and CCL19.	Breast cancer	Jing *et al* 79
Ad5-D24-RGD		*E1A* deletion; insertion of *RGD-4C* in the Ad5 virus. Combination with ECM-degrading proteases relaxin, elastase and MME.	Control of M1/M2 ratios; ECM remodeling.	Lung cancer	Lavilla-Alonso *et al* 42
Ad5/3-Δ24aCTLA4		Insertion of the cDNA sequence for an IgG2-type anti-CTLA4mAb to E3gp19K region of *E1A*-deleted Ad5/3 chimera (serotype 3 knob).	Inhibition of TRegs and increased CD8^+^/TReg ratios; Increased T cell activity.	Lung cancer; Ovarian cancer; Prostate cancer; Head and neck squamous cell carcinoma	Dias *et al* 74
Ad-TD-nsIL-12		Ad5 with *E1ACR2, E1B19K and E3gp19K* gene deletions encoding non-secreting *IL-12.*	Increased CD3^+^CD4^+^T cell infiltration; Reduced expression of inflammatory cytokines.	Pancreatic cancer	Wang *et al* 13
AdwtRGD-PH20		AdV with a fiber RGD motif expressing soluble *PH20* under the control of the major late promoter.	Decrease in tumor HA levels and wider areas of virus replication.	Prostate carcinoma	Guedan *et al* 78
AdCMVdelta24		24 bp deletion in *E1A.*	Decreased tumor-infiltrating TRegs and increased IFNγ-producing CD8^+^ T cells; Reprogrammed TRegs from an immunosuppressive to a stimulatory state.	Glioblastoma	Qiao *et al* 66
oAd/IL12/GM-RLX		*E1B19K and E1B55K* deleted AdV expressing *relaxin, IL-12, and GM-CSF*; combination with αPD-1.	Enhanced drug distribution; A higher level of I.T T cell infiltration (CD4^+^ and CD8^+^) and IFN-γ expression; Attraction of activated T cells and infiltration into poorly immunogenic tumors.	Gastric cancer; Pancreatic cancer	Jung *et al* 77
ICO15K-FBiTE		Deletion of 24 bp *E1A* region, RGD-modified fiber domain, expression of *FAP-CD3ɛ- targeting BiTE.*	Activation and proliferation of T cells; CAF targeting; Increased tumor T cell retention and accumulation.	Vulval epidermoid carcinoma; Lung cancer; Pancreatic cancer	de Sostoa *et al* 82
EnAd-CMV/SA-FAP-BiTE		Group B adenovirus Enadenotucirev expressing *αFAP/αCD3 BiTE.*	T cell activation and killing of autologous CAFs; Depletion of FAP^+^ fibroblasts; Increased IL-17A, IL-17F, IL-22, IFN-γ, and IL-10 expression; Repolarization of M2 ascites macrophages.	Colorectal cancer; Lung cancer	Freedman *et al* 83
OBP301		Human telomerase reverse transcriptase promoter element drives the expression of *E1A* and *E1B* genes linked with an internal ribosomal entry sequence.	Increased IFN-γ expression; Inhibition of angiogenesis.	Colorectal cancer	Ikeda *et al* 87
rAd-E1A		Expression of *E1A tumor suppressor* gene.	Induction of tumor cell apoptosis; Downregulation of VEGF and CD34; Reduced tumor blood vessel formation.	Hepatocellular carcinoma	Ye *et al* 86
EnAd‐CMV‐EpCAM BiTE		Group B adenovirus Enadenotucirev expressing *EpCAM BiTE,* regulate by CMV promoter.	Strong T‐cell activation; Depletion of EpCAM‐positive cells.	Ovarian cancer	Freedman *et al* 83
EnAd-CMV-BiTE/TriTE		BiTEs were CD3ε scFv, CD206-targeting nanobody or a folate receptor β-targeting scFv. Addition of anti-CD28 scFv or a second anti-CD3 scFv.	Activation and expansion of CD4^+^ and CD8^+^ T cells; Increased CD11b^+^CD64^+^ cells; Increased IFN-γ expression.	Colorectal cancer	Scott *et al* 48
CNHK500-mE		E1A gene controlled by the hTERT promoter and the E1b promoter is replaced by HRE; expression of endostatin.	Anti-angiogenic.	Hepatocyte cancer; Pancreatic cancer; Lung cancer; Breast cancer; Nasopharyngeal cancer; Cervical cancer; Gastric cancer	Su *et al* 94
CVB3	Coxsackie virus (positive-strand RNA genome)	---	Tumor cells express calreticulin and secreted ATP as well HMGB1; NK and granulocyte recruitment.	Lung cancer	Miyamoto *et al* 53
PVSRIPO	Poliovirus (positive-strand RNA genome)	Poliovirus genome carrying a heterologous IRES from HRV2.	Induction of extensive neutrophil infiltration; DC and T cell infiltration and activation.	Breast cancer; Prostate cancer	Holl *et al* 58
NDV-αCTLA4	Newcastle disease virus (negative-strand RNA genome)	NDV expressing anti-CTLA4 scFv.	TReg inhibition; Increased CD8^+^/TReg ratios.	Melanoma	Vijayakumar *et al* 73

AdV, adenovirus; BiTE, bispecific T cell engagers; CAFs, cancer associated fibroblasts; DC, dendritic cell; ECM, extracellular matrix; EnAd, enadenotucirev; FAP, fibroblast activation protein; GM-CSF, granulocyte-macrophage colony-stimulating factor; HMGB1, high mobility group box 1 protein; HSV, herpes virus; IFN, interferon; IL, interleukin; IRES, internal ribosomal entry site; I.T, intratumoral; mAbs, monoclonal antibodies; MDSC, myeloid derived suppressor cells; MeV, measles virus; MHC, major histocompatibility complex; mRNA, messenger RNA; MuV, mumps viruses; NDV, Newcastle disease virus; NK, natural killer; NO, nitric oxide; OVs, oncolytic viruses; PD-1, programmed cell death protein-1; PD-L1, programmed death ligand-1; TME, tumor microenvironment; TNF, tumor necrosis factor; TReg, regulatory T cell; VGF, viral growth factor; VSV, vesicular stomatitis virus; VV, vaccinia virus; WR, Western Reserve.

**Figure 1:**
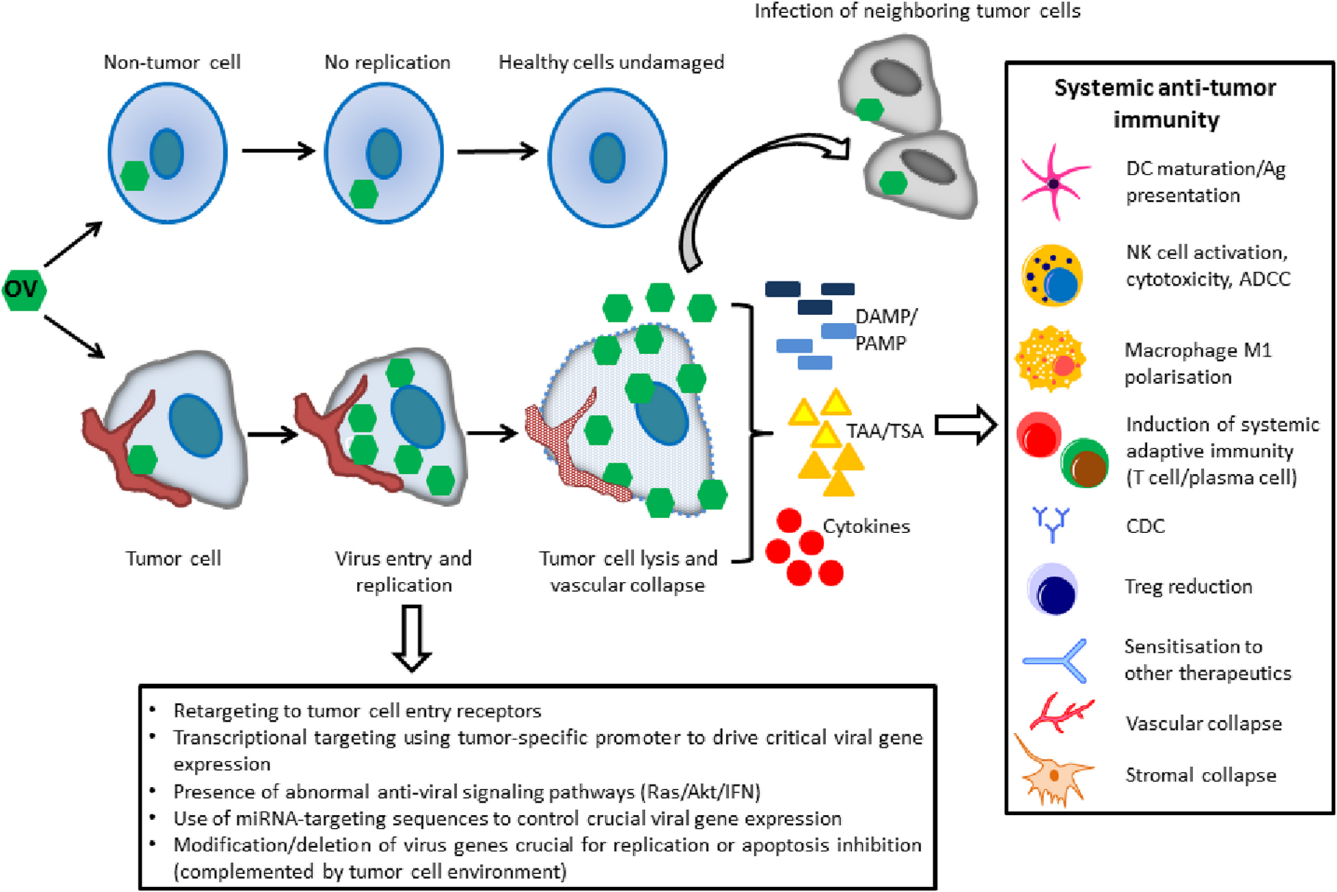
Actions of oncolytic viruses. Oncolytic viruses replicate selectively in tumor cells and selectivity is determined via receptor retargeting, transcriptional retargeting, abnormal antiviral signaling pathways in tumor cells, creating replication defects in healthy cells via tissue-specific miRNA expression or viral gene deletion or targeting anti-apoptotic pathways dysregulated in tumor cells. On generation of projeny virions, tumor cells are lysed and released projeny travel to infect neighboring tumor cells. Local inflammation that results from PAMP, DAMP, tumor antigen and cytokine expression consequent to OVT causes the development of a systemic antitumor immune response that can result in a long-term antitumor effect. ADCC, antibody-dependent cellular cytotoxicity; DAMP, damage associated molecular patterns; CDC, complement-dependent cytotoxicity; DC, dendritic cell; IFN, interferon; miRNA, micro RNA; NK, natural killer; PAMP, pathogen associated molecular patterns; TAA, tumor associated antigen; TSA, tumor specific antigen.

OV therapy (OVT) utilizes wild-type or genetically modified viruses that selectively replicate in tumor cells. OVs exert their effects through direct cell lysis, but more importantly by modification of the TME into an immune-rich environment that supports persistent tumor-specific immunity to kill primary, metastatic or recurring tumor cells (figure 1). OVs can be DNA or RNA-based viruses (table 2), many are wild-type strains that cause minimal or no disease in humans, including herpes virus (HSV), adenovirus (AdV), reovirus (RV), vesicular stomatitis virus (VSV) or the avian Newcastle disease virus (NDV). Others are attenuated strains such as the measles virus (MeV) Edmonston vaccine strain, mumps (MuV) viruses, or vaccinia virus (VV). Most have a degree of natural tropism towards tumor cells, commonly conferred by the natural sensitivity of viruses to virus-induced interferon (IFN) response pathways. Some OVs also rely on cancer cell over-expression of viral receptors or an innate selectivity for apoptosis-resistant cancer cells^6^ (figure 1). Advances in genetic engineering makes it possible to enhance or confer tumor specificity via rational gene deletion, use of tumor-specific promoters or micro RNA-targeting sequence to drive expression of essential viral genes in tumor cells only, as described previously (figure 1).^7^

**Table 2:** Comparison of the properties of the major DNA (top) and RNA (bottom) viruses used for development of oncolytic virotherapy platforms

DNA viruses	Vaccinia virus	Adenovirus	Herpesvirus	Parvovirus H1		
Natural host	Human (v)	Human	Human	Rat		
Genome	dsDNA	dsDNA	dsDNA	ssDNA		
Replication site	C	N	N	N		
Nuclear integration	–	+	+	+		
Cell receptor	Not needed	CAR	HVEM/nectin	Sialic acid residues		
Transgene capacity	+++	++	+++	–		
Delivery	i.t and i.v	i.t	i.t	i.t and i.v		
Hypoxia	+	–	+	n.k		
Pre-existing immunity	+	+++	+++	–		
Antiviral drugs available	+	+	+	–		
RNA viruses	Reovirus	Coxsackievirus	Poliovirus	Measles virus	NDV	VSV
Natural host	Human	Human	Human (v)	Human (v)	Bird	Human
Genome	dsRNA	ssRNA	ssRNA	ssRNA	ssRNA	ssRNA
Replication site	C	C	C	C	C	C
Nuclear integration	–	–	–	–	–	–
Cell receptor	Unknown	CAR/ICAM/DAF	CD155	SLAM/CD46	Unknown	LDLR
Transgene capacity	–	–	–	+	+	+
Delivery	i.t/i.v	i.t/i.v	i.t	i.t	i.t/i.v	i.t/i.v
Hypoxia	–	n.k	+	n.k	+	n.k
Pre-existing immunity	+++	+	+++	+++	–	–
Antivirals	–	–	–	–	–	–

C, cytoplasmic replication; CAR, coxsackie-adenovirus receptor; DAF, decay accelerating factor; dsDNA, double-stranded DNA; dsRNA, double-stranded RNA; HVEM, herpesvirus entry mediator; ICAM, intercellular adhesion molecule; i.t, intratumoral; i.v, intravenous; LDLR, low density lipoprotein receptor; N, nuclear replication; N, nuclear replication; NDV, Newcastle disease virus; n.k, not known; ssDNA, single-stranded DNA; ssRNA, single-stranded RNA; v, vaccine strain; VSV, vesicular stomatitis virus.

DNA viruses may have the strongest potential as OVs given their large capacity for genome modification and insertion (table 1) and their clinical success relative to RNA viruses.^8^ The first OV approved for clinical was H101 in China, an *E1B-55K* gene deleted recombinant Ad5 (human type-5 AdV).^9^ Selectivity of H101 is dependent on the unique ability of tumor cells expressing Y-box binding factor 1 to mediate export of late AdV RNAs in the absence of *E1B-55K*.^10^ More recent platforms have aimed to improve on the low efficacy of H101 using rational gene deletion strategies to enhance the antitumor effects. The ONCOS platform (Targovax) derives tumor specificity from a 24 bp deletion in the E1A protein that prevents pRB sequestration by the virus and thus prevents G1-S cell cycle progression and viral replication in healthy cells.^11^ Additionally, serotype switching of the AdV fiber knob, the main point of interaction between the virus and its receptor, from serotype 5 to 3, imparts an ability to infect cells via desmoglein 2 and CD46 receptors.^12^ The Ad-TD platform has three gene deletions to ensure selectivity and efficacy; E1ACR2, a 24 bp deletion as used in the ONCOS platform, E1B19K, an anti-apoptotic protein with dysregulated apoptosis pathways and E3gp19K that binds and sequesters major histocompatibility complex (MHC) I molecules, preventing immune-mediated virus clearance in healthy cells.^13^ HSV-based oncolytics are also proving clinically successful. T-VEC (imlygic) was approved worldwide from 2015 for treatment of refractory melanoma.^14^ Tumor specificity of this virus is derived by deletion of the *ICP34.5* and *ICP47* genes, which prevents virus-mediated inhibition of antiviral PKR pathways and MHC-I downregulation in healthy cells.^15^ More recently, Delytact (teserpaturev/G47), with a deletion in both copies of the γ*34.5* gene, the α*47* gene and the *ICP6* locus of HSV-1,^16^ has been approved for the treatment of patients with glioblastoma in Japan.

The potential of VV-based oncolytics is also emerging through late phase clinical trial results, with JX594 and derivatives (SillaJen) demonstrating varying degrees of efficacy in a number of tumor types. VVs have a natural tropism towards tumor cells. Their large size (~360 nm) prevents diffusion through healthy vasculature, but leaky tumor-associated vasculature with high vascular endothelial growth factor (VEGF) levels supports viral replication and allows virus to pass from the blood stream into tumor cells with ease^17^ and they show a degree of sensitivity towards type I IFN. The thymidine kinase (*TK)* gene deletion imparts tumor selectivity as cellular TK expression in cancer cells is constitutively higher than normal cycling cells.^18^ In many platforms, specificity may be supported by viral growth factor (*VGF),* ribonuclease reductase (*RR; F14L), N1L* or other gene deletions.^19 20^ Additionally since non-cancer cells use apoptosis as an antiviral defense mechanism, deletion of anti-apoptotic genes (SPI-1, SPI-2, F1L, and N1L) in VVs can increase selectivity towards tumor cells and enhance antitumor effects.^21–23^

## OVT and the TME

The TME is an actively supportive network comprizing stromal cells, immune cells, vasculature and altered metabolic pathways that promote tumor cell proliferation and metastasis. Recruitment and interactions of these components is a dynamic process, regulated by local cytokine and chemokine signaling networks that actively support continuous restructuring and growth at all stages of carcinogenesis.

Tumors are regarded as immunologically cold environments due to the low levels of tumor antigens and infiltration of tumor suppressive immune cells and signaling molecules. OVs are regarded as a powerful novel form of immunotherapy not only because of their ability to specifically target and lyse tumor cells,^24^ but most importantly their capacity to break down the immune suppressive environment to create an immunologically ‘hot’ environment that promotes long-term tumor-specific immunity to provide surveillance against relapse^25^ (figure 1). Multiple clinical studies have demonstrated that induction of tumor specific immunity is a key element affecting prognosis and survival of patients with tumor.^26^ OV-mediated remodeling of the immunosuppressive TME is able to create an environment that favors influx and activation of antitumor immune cells, promoting tumor elimination.

## The effect of OVT on the immune milieu within the

### OVT and the dendritic cell–T cell axis

A critical characteristic of OVs is their ability to induce immunogenic cell death that causes the release of damage associated molecular patterns (DAMPs, including calreticulin, ATP, uric acid, heat shock proteins, high mobility group box 1 protein (HMGB1)), pathogen associated molecular patterns (PAMPs, including double-stranded DNA, double-stranded RNA, single-stranded RNA, glycoproteins, lipoproteins and viral membrane components) and cytokines (IFN-γ, IFN-α, tumor necrosis factor (TNF)-α, interleukin-1 (IL-1), IL-6, IL-8, IL-12).^27^ These molecules act as danger signals that activate dendritic cell (DC) infiltration and maturation.

DCs are a rare occurrence in tumors, but represent powerful, professional antigen-presenting cells (APCs) that connect the innate and adaptive immune system. Immature DCs have a strong capacity for migration, while mature DCs activate T cells by expressing speciﬁc T cell adhesion and co-stimulatory molecules.

**Figure 2:**
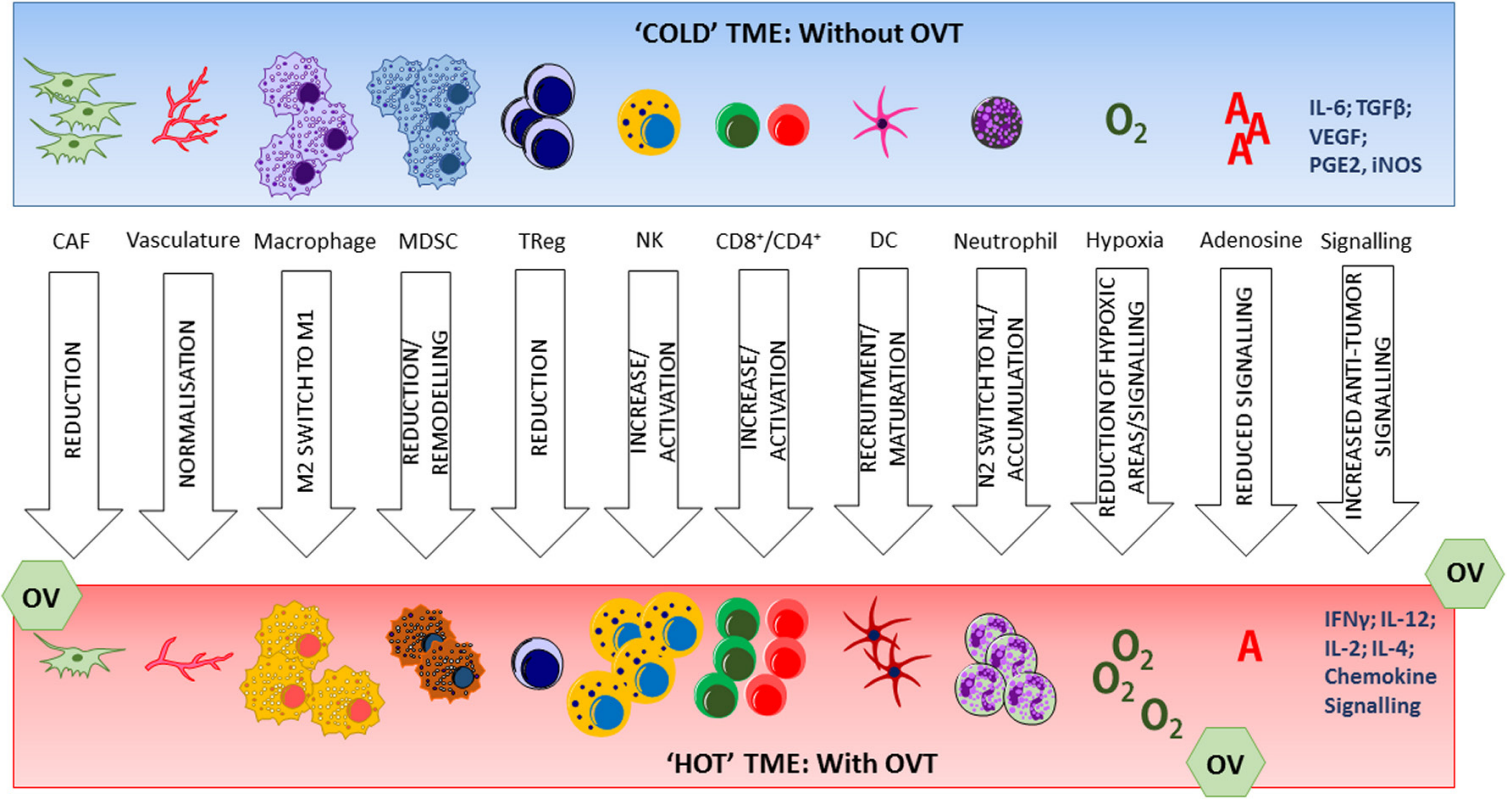
The tumor microenvironment prior to and in response to oncolytic virotherapy. The ‘cold’ TME is comprised of a dense stroma and immunosuppressive cells. Antitumor immune cell infiltration is rare. Treatment with OVT can cause local vascular collapse, tumor cell death and remodeling of the suppressive immune and metabolic environment to one that favors immune-mediated tumor clearance (‘hot’). CAF, cancer associated fibroblast; DC, dendritic cell; IFN, interferon; IL, interleukin; MDSC, myeloid derived suppressor cells; NK, natural killer; OV, oncolytic viruses; OVT, OV therapy; TGF, transforming growth factor;.TME, tumor microenvironment; TReg, regulatory T cell; VEGF, vascular endothelial growth factor.

The activation status of DCs is critical to their function. In the absence of danger signals, or in a suppressive cytokine environment, DCs are unable to mature fully, consequently inducing T cell tolerance to tumor antigens by downregulation of their antigen presentation capacity (ie, downregulating CD40, CD80, CD86, IL-12 while upregulating programmed death ligand-1, T cell immunoglobulin and mucin domain-containing protein 3, IL-10 and indoleamine 2,3-Dioxygenase 1).^28^ The regulation of DC populations in the TME is crucial for controlling effective antitumor immunity and OVs are able to not only provide novel tumor antigens to DCs as a result of their oncolytic effect, but also prime the TME to support DC infiltration and maturation such that the DC antigen presentation pathway is effective at T cell activation (figure 2). Moreover, upregulation of antitumor cytokine expression within the TME consequent to OV infection also increases the infiltration and activation status of CD4^+^ and CD8^+^ T cells into the TME that initiate adaptive antitumor immune responses (figure 2). Increased expression of antitumor cytokines within the TME has wide-ranging effects on tumor cells (restoration of MHC-I antigen presentation pathways), immune cells (repolarization or inhibition of inhibitory cells), and other elements of the TME (vasculature, the metabolic environment, cancer associated fibroblasts (CAFs) and the ECM). These alterations may act to support ongoing activation of innate and adaptive antitumor immune responses and long-term tumor suppression.

#### Increasing the power of OVT to induce DC/T cell activation

While OVT has a profound immunological effect within the tumor, clinical use of H101 demonstrated that OVT alone may not be powerful enough. More recent investigations have focused on arming OVs with therapeutic payloads, commonly monoclonal antibodies (mAbs), cytokines or chemokines. OV-mediated delivery of pro-immune cytokines directs expression to the TME, localizing immune reaction and limiting toxic side effects. Additionally, the self-perpetuating nature of replicating OVT ensures a constant supply of cytokines. Granulocyte-macrophage colony-stimulating factor (GM-CSF) is a commonly investigated cytokine in this regard. GM-CSF plays an important role in the recruitment, activation and maturation of monocytes and DCs into potent antigen presenting cells. Preclinical data has demonstrated that the GM-CSF-armed HSV T-VEC (imlygic) had a stronger abscopal effect compared with unarmed virus after intratumoral injection, demonstrating the value of GM-CSF in activating systemic immune responses that can target tumors at multiple sites around the body.^29^ JX594, a GM-CSF-armed VV has been analyzed in a number of clinical trials. While the safety of this virus has been established, clinical evidence of efficacy is lacking. The actions of GM-CSF are largely restricted to myeloid cells and in some cases GM-CSF been shown to promote tumor progression,^30^ thus alternative cytokines with a more pleiotropic effect on the immune system may be preferable. IL-12, a master regulator of antitumor immune responses, is one of a number of cytokines to be investigated as an alternative payload. IL-12 plays vital roles in DC, natural killer (NK) and T cell maturation and increases IFN-γ levels in the TME. It has also been shown to inhibit myeloid derived suppressor cells (MDSC) and regulatory T cell (TReg) activities, induce M1 polarization of macrophages and plays in important role in the inhibition of angiogenesis. A number of OVs have been armed with IL-12 and pre-clinical data shows encouraging antitumor effects (reviewed recently in.^31^ Of these, an oncolytic HSV with a deletion of the *ICP6 RR* gene to restrict replication to tumor cells (HSVΔG47-IL-12) is one of the most advanced and is currently being investigated in clinical trials for glioma.^32^ A major concern regarding IL-12 is that systemic accumulation of IL-12 can result in the rapid development of lethal inflammatory syndrome.^13^ OV-mediated delivery of IL-12 is an elegant way in which to restrict delivery and expression to the tumor, preventing systemic toxicity, however while intratumoral (I.T) delivery of IL-12-armed vectors has been demonstrated as safe, intravenous delivery of the same vectors is associated with IL-12-mediated systemic toxicity. A triple-deleted oncolytic AdV expressing a mutant form of IL-12 that is not secreted from infected cells (Ad-TD-nsIL12) was demonstrated to circumvent these toxicity issues and maximize the therapeutic potential of OV-IL-12.^13^

Several other antitumor cytokine or chemokine molecules are being investigated in the context of OVT with the hope of improving robust antitumor efficacy. IL-2, a potent T cell activator, has long been researched but can also evoke considerable systemic toxicity. Recently, Lui *et al* reported that the delivery of a modified IL-2 with tumor cell-surface restricted expression by a Western Reserve (WR) strain VV deleted for *TK* and *VGF* genes (vvDD) was safe and effective at mediating tumor regression in murine cancer models.^33^ IL-21 has been evaluated as a pleiotropic cytokine with a strong safety profile in the context of a gene deleted, intravenous-delivered VV platform.^34^ Additionally, VV armed with IL-36*γ* induced infiltration of DC and T cell differentiation to promote antitumor adaptive immunity.^35^ VSV-IFN-β-NIS (Voyager V1) is currently under clinical evaluation as a systemically deliverable OV for multiple tumor types, the IFN-β payload included as an agonist of the intracellular STING pathway that results in type I IFN production and thus activation of innate and adaptive immune responses.^36^ OVT can also affect the chemokine environment to induce immune-cell homing to the TME.^23^ Chemokine-arming strategies (CXCL11, CCL5) are being investigated in an attempt to enhance this effect and these may prove useful vectors for coadministration with chimeric antigen receptor-T therapies to promote their infiltration.^37 38^ Suicide gene- or pro-drug-arming is a further strategy to enhance therapeutic efficacy. Suicide genes promote the killing activity of OVs and include TNF-related apoptosis-inducing ligand. VV (VG9, Tian Tan strain Guang 9) armed with cytosine deaminase allows for the conversion of the non-toxic prodrug 5-fluorocytosine into drug 5-Fluorouracil.^39^ It is worth noting that a balance must always be struck between transgene selection and viral activity, as payloads that cause direct cell death will limit viral propagation in tumors and negate oncolytic and immune-stimulatory effects associated with OVT.

### OV and macrophages

Macrophages are characterized by strong plasticity, with M1 and M2 populations representing the extremes of a continuum of different states. The presence of M2 macrophages in the TME is a strong prognostic indicator for decreased overall survival in a number of cancers.^40^ OV-treatment has been shown to cause the repolarization of immune-suppressive I.T M2 macrophages towards an M1 phenotype that express pro-inflammatory cytokines and chemokines including IFN-γ, CXCL10, IL-6, IL-2, IL-12, IL-21 among others that reinforce the antitumor immune environment. This effect has been noted with several OVs including GLV-1h68^41^ (GL-ONC1, Lister strain VV) and Ad5-D24-RGD^42^ (tumor-retargeted oncolytic AdV), VSV,^43^ HSV,^44^ RV,^45^ paramyxoviruses^46^ (MeV and MuV) and VV-IL21,^34^ demonstrating OVT as an effective mechanism by which to control M1/M2 ratios in the TME. A recent report revealed that treatment with an *ICP34.5*-deleted HSV vector (HSV1716) was able to not only promote M1 polarization of macrophages, but macrophages could also take up, replicate and release HSV1716 into the TME, providing a viral amplification route and promoting the oncolytic effect of the treatment.^47^ Additionally, treatment with both GL-ONC1 and Ad5-Δ24-RGD have been shown to induce metallelastase production by macrophages in colorectal cancer models. Metallelastase has proteolytic activity against many ECM components, dissolving the physical barrier to immune and therapeutic delivery, increasing I.T viral spread, viral oncolysis and inhibiting tumor angiogenesis.^41 42^

Recently an OV/bispecific T cell engagers (BiTE) strategy to directly target M2 macrophage populations has been reported based on the differential surface marker expression of M2 versus M1 macrophages. BiTEs are scFv antibodies targeted against CD3 on the T cell and the tumor antigens on tumor cells, resulting in the formation of a pseudo-immunological synapse between CD3^+^ T cells and target cells. However, when delivered systemically, toxicity and tumor penetration issues associated with BiTEs have emerged, thus it is becoming more common to develop OV-based delivery strategies for these agents. An αFRβ/αCD3 BiTE was encoded in enadenotucirev (EnAd), the group B oncolytic AdV, to target folate receptor expression common to M2 macrophages. Analysis of efficacy in human malignant ascites demonstrated this vector caused T cell activation and M2 depletion.^48^ A further issue to consider in is the direct effect of macrophages on OVT clearance that can prevent effective systemic delivery of OVT. Recently, Ferguson *et al* reported that systemic delivery of VV is hampered by macrophage uptake, but that transient inhibition of macrophage phagocytosis using a pharmacological inhibitor allowed intravenous-delivered VV to reach the tumor in therapeutic quantities.^49^

### OV and NK cells

NK cells play a critical role in tumor elimination, providing both direct cytotoxic functions and remodeling the environment in the TME. OVT-activated DCs provide type I IFN, IL-12 and IL-18 to the TME that enhance the cytotoxicity of NK cells.^50^ Production of other signaling mediators including IL-15 or IL-21 by myeloid cells in the TME enhance this effect further.^50^ OVT mediates NK recruitment into the TME by increasing production of chemokines and creates the cytokine milieu to promote and sustain NK activation (figure 2). Additionally, it has been reported that OVs modulate the expression of activating and inhibitory NK ligands, including MHC-I, on the cancer cells.

A critical antitumor role of OVT-stimulated NK cells has been noted in many studies using many different OVTs including HSV,^51^ RV,^52^ CV^53^ and VV^23^ among others. The recent report of an *N1L* deletion in the *TK*-deleted VV Lister strain demonstrated improved antitumor NK responses to VVΔTKΔN1L compared with VVΔTK, demonstrating that rational alteration of viral backbones by modification of genes critical for controlling immune responses to viral infection can create more potent OVTs.^23^ It was demonstrated that VSV infection sensitized tumors to NK cell recognition and killing via IL-28 activation and a subsequent VSV-IL28 therapy was even more effective at NK cell induction.^54^ A further important effector mechanism of NK cells involves antibody-dependent cellular cytotoxicity (ADCC), whereby NK cells target cell-surface bound antibodies to induce cell lysis. Niemann *et al* have developed a novel approach to enhance oncolytic AdV therapy using a bifunctional molecular with a tumor-specific ligand and AdV hexon domain to target post-AdV OVT neutralizing antibodies (nAB) to the tumor, enhancing NK-mediated antitumor ADCC responses.^55^ It should be considered however, that NK cells are primary mediators of antiviral immunity, thus their activation by OVT may reduce and limit OV spread. Indeed, a recombinant VSV vector, rVSV-UL141, that downregulates the NK activating receptor CD155, enhances its oncolytic capacity by inhibiting NK and NKT.^56^

### OV and neutrophils

While there is little data available on the phenotypic characterization of neutrophils in clinical tumors post-OVT, many OVTs have demonstrated a pre-clinical ability to induce neutrophil accumulation within the tumor that is associated with therapeutic efficacy. Pre-clinical evidence suggests that TGF-β-polarized N2 neutrophils have a pro-tumor, anti-immune effect, while type-1 IFN-polarized N1 neutrophils have an antitumor, pro-immune effect. Antitumor effects of N1 neutrophils have been attributed to the recruitment and activation of CD8^+^ T cells and production of cytokines including IL-12, TNF-α, GM-CSF and VGF. They also attract macrophages, DCs, NKs and T cells via IL-1β and MIP-1α production, mediate direct cytotoxicity towards tumor cells and mediate mechanical disruption of tumor vasculature. Infection with both VV-based and VSV-based viruses resulted in antitumor effect via a neutrophil-induced reduction in blood flow to tumors and consequent apoptosis of non-infected tumor cells.^57^ PVSRIPO, a recombinant poliovirus vaccine strain carrying a heterologous IRES (internal ribosomal entry site) from human rhinovirus induces extensive neutrophil infiltration in human prostate and breast cancers associated with therapeutic effect.^58^

### OV and MDSC

MDSCs are highly immunosuppressive cell populations in the TME, mediating inhibition via several mechanisms including depletion of arginine that causes T cell dysfunction and apoptosis, secretion of immunosuppressive cytokines, production of reactive oxygen species, cysteine depletion, modification of the T cell receptor and induction of TReg cells.^59^ Accumulation of MDSC consequent to viral infection has been shown in a number of tumor models,^60 61^ however some OV have been demonstrated to remodel MDSC populations in the TME. The oncolytic VV GLONC-1 is able to convert MDSCs to tumor killing phenotypes by increasing nitric oxide (NO) production from these cells.^61^ The in situ conversion of protumor to antitumor MDSCs contributes to the therapeutic effect of GLONC-1 although the exact mechanisms remain unclear. It is possible the transient production of NO, that is, toxic to T cells, either inhibits early viral clearance or precedes T cell infiltration into tumors. RV inhibits the immunosuppressive activity of MDSC in a TLR3-dependent manner, despite virus-mediated upregulation of IL-6 and TNF-α that can promote MDSC activity.^62^ Interestingly infection of MDSC with VSV, to allow for systemic delivery of VSV by harnessing the tumor-localizing properties of MDSC, converted tumor-promoting MDSCs to tumor-suppressive phenotypes and these populations acted synergistically with VSV to eliminated established tumors.^63^ Strategies to facilitate OV-mediated MDSC reduction in tumors include hydroxyprostaglandin dehydrogenase (HPGD) enzyme arming of VV. HPGD inactivates PGE2 and reduces the presence of MDSCs in the TME,^64^ sensitizing tumors to the immunotherapeutic effects of VV. Similarly, VV-mediated expression of a CXCR4 agonist inhibited the effects of CXCL12 on angiogenesis and recruitment of MDSCs to the TME in ovarian cancer models.^65^

### OV and TRegs

TRegs are generally characterized by expression of the transcription factor Foxp3 and their strong immunosuppressive activity. AdCMVΔ24 infection was demonstrated to decrease I.T TReg levels.^66^ Infection-induced TReg loss has also been shown for VV, with transient reduction identified consequent to pathogen-induced expansion of effector T cells, which decreases availability of IL-2 critical for maintenance of TReg activity.^67^ More recently, Depaux *et al* reported that oncolytic VV can directly infect tumor infiltrating TReg cells in murine models of head and neck cancer, resulting in depletion and successful antitumor effects after treatment.^68^ To improve therapeutic effect, combination agents are being examined. VSV requires PC61 (α-CD25 antibody) to reduce TRegs and prevent TReg-mediated inhibition of NK cells. Targeting chemokine or cytokine signaling is also showing promise; CXCL12 induces I.T localization of TRegs. CXCL12 inhibition, using VV armed with a CXCR4 agonist, reduced TReg levels^65^ and the HSV vector G47Δ-mIL12 decreased both TRegs and angiogenesis.^69^ Interestingly, it has been demonstrated that I.T TRegs overexpress CTLA4 relative to CD4 and CD8 cells^70^ and the therapeutic effect of immune checkpoint inhibitors (ICI) has been shown in part to be due to their ability to deplete TRegs in an FC-dependent manner via ADCC,^71^ increasing I.T CD8^+^/TReg ratios. An MV expressing α-CTLA4 or α-PD-1 was demonstrated to inhibit TRegs and increase CD8^+^/TReg ratios in the melanoma TME.^72^ Similar findings were reported for NDV-αCTLA4 and AdV-αCTLA4.^73 74^

## OVT remodeling of the stromal, vascular and metabolic components of the TME

In addition to malignant and immune cells, the TME comprises many other cell types, recruited by tumor-released cytokines or chemokines and transformed from in situ cells to tumor-associated cells that support tumor development and ongoing growth.

### OVs and the tumor stroma

The stroma of the TME includes the ECM and CAFs that orchestrate many aspects of tumor biology and limit therapeutic efficacy by preventing treatment infiltration. The ECM is an integral part of the cancer stem cell (CSC) niche and ECM receptors have been shown to aggregate CSCs^75^ and induce drug resistance.^76^ A number of OV-mediated approaches have been described that directly target the stroma with the aim of eliciting durable ECM remodeling to improve therapeutic distribution. An oncolytic AdV vector expressing relaxin in combination with immune modulators (oAd/IL-12/GM-RLX) was demonstrated to degrade the ECM, enhance penetration of mAB therapeutics and promote durable antitumor responses in models of pancreatic cancer.^77^ Oncolytic AdV expressing PH20 hyaluronidase demonstrated hyaluronan destruction in the ECM that facilitated OV dissemination.^78^ Importantly, this treatment left the protein matrix of the TME intact, thus while OV penetration was facilitated, metastasis of tumor cells from the tumor was not.

MV retargeted to urokinase receptors, abundant on both CAFs and tumor cells, inhibited the growth of breast cancer and importantly altered gene expression patterns associated with angiogenesis and inflammation.^79^ A key characteristic of CAFs is the expression of fibroblast activation protein (FAP), upregulated in many cancers.^80^ An oncolytic VV, Western reserve double-deletion (WRDD), expressing αFAP/αCD3 BiTE demonstrated effective CAF targeting and antitumor effect in melanoma.^81^ ICOVIR15K, a Δ24 oncolytic AdV armed with αFAP/αCD3 BiTE increased accumulation of T cells and decreased FAP expression in models of lung and pancreatic cancer^82^ and EnAd expressing αFAP/αCD3 BiTE demonstrated therapeutic effect via activation of tumor resident T cells towards CAFs and reduction of CAF-mediated immunosuppression, including repolarization of M2 ascites macrophages.^83^ A recent report suggests that the interaction of CAFs with tumor cells can result in upregulation of IFN-related transcriptional programs that inhibits OVT activity, suggesting that combining OVT with IFN-inhibition may be preferable to support OVT antitumor activity.^84^ Conversely there is evidence that CAFs may actually promote OV infection. Ilkow *et al* demonstrated that CAFs have an increased sensitivity to OV infection compared with normal fibroblasts. TGF-β production in CAFs dampens their antiviral responses, sensitizing them to infection by a number of OVs, including VV, Marbara MG1 and VSVΔ51. In turn, CAFs produce FGF2 that induces RIG-1 expression in tumor cells, impairing innate immune responses in the tumor cells and promoting OV infection. Thus, they proposed that cross-talk between CAFs and tumor cells creates a niche of OV-sensitive cells within the TME.^85^

### OVs and tumor vascularization

Targeting angiogenic pathways is a commonly used antitumor clinical approach, however resistance to these agents is common and rapid in onset. Many OVs exhibit direct anti-vascular properties. The E1A protein of AdV downregulates VEGF expression.^86^ An oncolytic AdV (OBP-301, Telomelysin), in which the human telomerase reverse transcriptase promoter element drives expression of *E1* genes was reported to mediate vascular collapse through activation of immune-mediated anti-angiogenic factor production.^87^ VSV targeting of I.T blood vessels induces clot formation and inflammation within tumor blood vessels.^88^ VV has also been demonstrated to exert powerful anti-angiogenic effects. Dysregulated epidermal growth factor receptor (EGFR)/Ras signaling pathways and increased levels of VEGF in tumor vasculature act to naturally support VV infection that results in vascular leakage and collapse.^89^ Vascular leakage, rather than normalization that is often associated with anti-angiogenics, facilitates systemic delivery of VV from vasculature to tumor cells.^90^ Arming strategies to address neovascularization in the tumor have been explored. Endostatin and angiostatin arming has been demonstrated to improve vascular collapse in VV,^91^ HSV,^92^ MV^93^ and AdV-based systems.^94^ Similarly, α-VEGF antibody arming strategies have been explored in VV and AdV systems that can result in decreased microvessel density.^95 96^ IL-12, CXCR12 and CXCR4 have well-established anti-angiogenic properties.^65^ While OVs may be powerful agents to target tumor angiogenesis, blood vessel perfusion recovers as the virus is cleared, demonstrating a need for multiple injections or sequential combination with alternative agents.^90^

### OVs and the metabolic environment

Metabolic reprogramming in cancer cells enables sustained proliferation and prevents induction of antitumor immune responses. The hypoxic response induced in tumors mediates therapeutic resistance and can be refractory to viral therapy. AdV types 5, 3 and 11 are attenuated under hypoxic conditions.^97^ On the other hand, hypoxia-induced upregulation of VEGF has been shown to augment VV infection^17^ and hypoxia-induced *GADD34* upregulation improves replication of HSV^98^ suggesting these viruses as effective therapeutics to eliminate resistant hypoxic fractions in tumors. VSV replication is comparable in hypoxic and normoxic conditions.^99^ RV reduces HIF-1α expression in hypoxic tumor fractions, contributing to the overall oncolytic effect by reducing transcription of HIF1α-responsive genes including *VEGF* and those responsible for maintaining CSC phenotypes.^100^ Many groups have attempted to target hypoxic fractions using hypoxia-specific promoters, however these are often less powerful drivers of gene expression compared with constitutive viral promoters. HIF-1α is a strong inducer of CD39, CD38 and the A2Rs that are key mediators of extracellular adenosine signaling in the TME, thus OV-mediated elimination of hypoxic fractions can also effect changes in the adenosinergic environment. Adenosine signaling in the TME activates intracellular cyclic adenosine monophosphate and is associated with profound immune suppression.^101^ OVs can also be used in combination with small molecule inhibitors or mAbs targeting these receptors, many of which are being investigated clinically^102^ to reduce the accumulation and activity of extracellular adenosine. In both hypoxic and normoxic tumor cells, aerobic glycolysis becomes the main driver of energy production and constitutes a classical hallmark of cancer.^103^ Glycolysis is induced in many cells in response to viral infection and as such, the altered metabolic pathways may support viral replication, a finding confirmed for HSV.^104^ Antiviral IFN responses also require glycolysis for activity, thus co-treatment with inhibitors of this pathway may advantageously reverse metabolic reprogramming and better support viral replication, oncolysis and induction of antitumor immune responses via lactate reduction.^105^ Given the role of tumor metabolism in suppressing effective antitumor immunity, OV monotherapy may be insufficient to promote T cell activation in this environment. Rivadeneira *et al* have recently described an oncolytic VV expressing leptin, with potent metabolic reprogramming functions that showed efficacy in murine melanoma models via oncolysis, stimulation of T cell infiltration and leptin-mediated reprogramming of T cells to support their activity.^106^ These results demonstrate a powerful novel approach to antitumor therapy, by directly targeting metabolic pathways to enhance OV-mediated induction of immune responses.

## Future perspectives

OVTs provide a powerful mechanism to target multiple elements of the TME simultaneously and thus represent a therapeutic with the potential to overcome therapeutic resistance and recurrence. Research uncovering the complex nature of the TME is providing the field of OVT with multiple opportunities to tailor novel therapeutics towards these elements to provide more robust therapeutic opportunities. Pre-clinical development must be rationally approached based on the ever growing wealth of information available regarding specific tumor TMEs. The selection of virus strain dictates efficacy against different elements of the TME, the ability to deliver additional payloads to enhance therapeutic effect and options for routes of delivery. The DNA viruses HSV and AdV have demonstrated clinical potential and have capacity to deliver additional payloads, however delivery of these viruses is currently mainly restricted to I.T delivery routes. While this method is clinically feasible for some tumor types and may avoid potential impact of nAB on intravenous delivery of oncolytic viruses, I.T delivery could result in limited viral distribution along the needle track only. Mechanisms for intravenous delivery are being urgently sought to allow for simultaneous targeting of primary tumors and metastatic or disseminated cells which could produce a greater therapeutic effect. VV in this regard may be a more attractive candidate. It has a wide tropism, can produce a particular form of virion after infection, EEV (extracellular enveloped virion), which can escape from nAB and complement clearance, can deliver multiple payloads without compromise of replication and cytotoxicity, can target multiple elements of the TME directly and effective intravenous delivery has been demonstrated even in the face of pre-existing antiviral nAB.^107 108^ To ensure associated efficacy, higher dosing or combination of I.T and intravenous delivery routes may be considered. Research continues into optimization of viral delivery processes, investigating carriers that can avoid immune recognition and sequential use of genetically distinct viruses to improve efficacy of multiple injections. Additionally, imlifidase, an endopeptidase able to degrade circulating IgG, has been shown to decrease anti-Adeno-associated virus (AAV) nAB and enhance AAV transduction efficiency in pre-clinical studies,^109^ this strategy may apply to intravenous delivery of VV or other oncolytic viruses.

With a wealth of safe viruses available to researchers, in-depth analysis of tumor responses to OVs or payloads is also now required in order to select the most appropriate virus for treatment of particular tumors. In this regard, biomarkers that can predict efficacy of treatment with different OVs are being sought. Zloza *et al* reported that immunoglobulin-like transcript 2 could be used as a therapeutic biomarker in patients treated with oncolytic VV^110^ and HMGB1 has been suggested as a predictive and prognostic biomarker for treatment with oncolytic AdV.^111^ Payload choice must address the needs of the TME, and will also be critical to mediate the fine balance between antiviral and antitumor immunity. Additionally, while OVT can effectively confine initial therapeutic responses to the tumor, multiarmed or systemically delivered vectors will need to be carefully constructed to ensure tumor-specific expression and prevent systemic toxicity.

‘OVT enhances other therapeutic approaches and clinical trials with OVTs are underway investigating their combination with other agents, including ICIs. ICIs represent a turning point in the cancer immunotherapy field, but only a proportion of patients currently benefit from ICI therapies. OVT could sensitize tumors to ICIs, augmenting therapeutic responses and overcoming primary therapeutic resistance although the phase III study of T-VEC in combination with pembrolizumab failed to meet its progression-free survival primary endpoint as presented at the ESMO Congress 2021 (Abstract 1037O). This warrants further investigations to identify which group of patients may get benefit from the combination. Tumor-selective OVs are likely to provide a means to improve multiple therapies by targeted destruction of the TME to enhance therapeutic infiltration and by modification of the soluble environment to promote infiltration and activation of the immune system. The focus of clinical trials now must be on optimization of dosing and combination regimes to provide the most efficacious regimen.’
